# Supplementary material for: The genome sequence of the Cranberry Fritillary, Boloria aquilonaris (Stichel, 1908) (Lepidoptera: Nymphalidae)
Source: Wellcome Open Res. 2025 Jul 28;10:378. [Version 1] doi: 10.12688/wellcomeopenres.24620.1 (PMC12334919; doi:10.12688/wellcomeopenres.24620.1)
Supplement: Peer review history

# Reviewer report: Reviewer response for version 1

**DOI:** 10.21956/wellcomeopenres.27130.r127938

**Published:** 2025-08-12

**Authors:** Doga Cedden

**Affiliations:** 1 University of Göttingen, Göttingen, France

Wymann et al. report a high-quality genome assembly from *Boloria aquilonaris*. The background information is sufficient, and the figure showing the specimen’s wings is adequate. The authors provide detailed quality control, with well-described methods. The sequencing data and genome assembly are publicly accessible through the provided accession number.

 My only comment concerns Figure 3. I recommend removing INSDC accession: OZ185200.1 (W) to avoid potential confusion.

Are sufficient details of methods and materials provided to allow replication by others?

Yes

Is the rationale for creating the dataset(s) clearly described?

Yes

Are the datasets clearly presented in a useable and accessible format?

Yes

Are the protocols appropriate and is the work technically sound?

Yes

Reviewer Expertise:

I work on coleopteran pests using RNA-seq and RNAi.

I confirm that I have read this submission and believe that I have an appropriate level of expertise to confirm that it is of an acceptable scientific standard.

# Reviewer report: Reviewer response for version 1

**DOI:** 10.21956/wellcomeopenres.27130.r127935

**Published:** 2025-08-08

**Authors:** Thomas Brown

**Affiliations:** 1 Leibniz IZW (Berlin) & ERGA/BGP, Leibniz, Germany

Wyman et al present a high-quality chromosome-level assembly of a European butterfly, *Boloria aquilonaris*. The methods of sampling, sequencing and assembling the individual and genome are state-of-the-art and the resulting assembly is of a quality that will likely be of use to the scientific community.

 Many details are given in the introduction regarding the morphological features and habitats of the Cranberry Fritillary, as well as detailing some current threats to its distribution, however there are no details given on why producing a genome for such a species will be of particular use in investigating certain features or assisting in its protection. At least a reference or link to what Project Psyche intends to do with such a genome would help the reader understand the motivation for sequencing this butterfly in particular.

 The metadata, data and genome assemblies are well reported and easy to find in the relevant databases and references.

Are sufficient details of methods and materials provided to allow replication by others?

Yes

Is the rationale for creating the dataset(s) clearly described?

Partly

Are the datasets clearly presented in a useable and accessible format?

Yes

Are the protocols appropriate and is the work technically sound?

Yes

Reviewer Expertise:

Genome assembly, genome annotation, FAIR (meta)data

I confirm that I have read this submission and believe that I have an appropriate level of expertise to confirm that it is of an acceptable scientific standard.

# Reviewer report: Reviewer response for version 1

**DOI:** 10.21956/wellcomeopenres.27130.r127940

**Published:** 2025-08-08

**Authors:** Noah H Rose

**Affiliations:** 1 University of California San Diego, San Diego, California, USA

This manuscript describes the sequencing and assembly of a chromosome-scale reference genome for the Cranberry Fritillary. The authors use a combination of PacBio and Hi-C approaches – this combination is well suited to their goals. The methods are state-of-the-art and clearly described, and the assembly appears to be of high quality. This will serve as a useful resource to the insect genomics community.

Are sufficient details of methods and materials provided to allow replication by others?

Yes

Is the rationale for creating the dataset(s) clearly described?

Yes

Are the datasets clearly presented in a useable and accessible format?

Yes

Are the protocols appropriate and is the work technically sound?

Yes

Reviewer Expertise:

Ecological genomics, evolutionary genetics, adaptation

I confirm that I have read this submission and believe that I have an appropriate level of expertise to confirm that it is of an acceptable scientific standard.
